# Are Estrogens Involved in the Earlier Onset of Psoriasis in Girls? Comment on Cassalia et al. How Hormonal Balance Changes Lives in Women with Psoriasis. *J. Clin. Med.* 2025, *14*, 582

**DOI:** 10.3390/jcm15010084

**Published:** 2025-12-23

**Authors:** Francisco Urbina

**Affiliations:** Independent Researcher, Algeciras 583, Las Condes, Santiago de Chile 7580258, Chile; fcourbina@hotmail.com

**Keywords:** psoriasis, early-onset psoriasis, psoriasis epidemiology, estrogens, puberty, metabolic syndrome

I read with interest the review of Cassalia et al. [1] about hormonal balance in women with psoriasis and how variations in estrogen levels could influence the improvement or worsening of the disease during menstrual cycle, pregnancy, and menopause. However, no comment was made on this during puberty or in the years preceding it regarding the development of psoriasis. Although not absolutely definite, there are numerous studies that have shown an earlier age of onset of psoriasis in females compared to males, especially around puberty. So far, there is apparently no explanation for this, despite it having been reported in different populations and races. Unfortunately, many valuable studies about childhood psoriasis do not analyze these variables in depth, are incomplete, or do not provide adequate data for comparison.

Almost a century ago, Hoede from Germany found early onset of psoriasis in females based on 959 cases, and likewise, Romanus from Sweeden, in a follow-up of 1417 patients, noticed that 50% of the males patients were affected before 19 years of age and 75% before 26 years, while 50% of women were affected before 12 years and 75% before reaching 19 years of age [2]. This female predominance in early-onset psoriasis has been subsequently described in various countries, considering that girls with psoriasis usually outnumber boys during childhood or adolescence, that their average age of onset occurs earlier than that of boys, and that higher prevalence rates are observed in the female sex (Table 1).

A systematic review published in 2021 that included the prevalence of psoriasis by gender in children [37] (eight studies, in Supplementary Material, Table S5) showed that in six of the collected references with age ranges between 0 and 18 years, the lifetime prevalence was somewhat higher in females in all six studies; but analysis of the remaining two publications—with age ranges between 12–20 and 12–17 years—showed that the prevalence point was five times higher in females with respect to males in both [37]. These facts suggest that some precipitating factor for psoriasis may be acting in girls in those age ranges. Recently, a large epidemiological study conducted in Poland that included 639,662 patients with psoriasis also showed a slight higher prevalence in girls between 0 and 9 years of age, which abruptly rose noticeably in the following next intervals: boys (0.88) and girls (1.34) between 10 and 14 years old and 1.42 versus 1.99, respectively, between 15 and 19 years. The difference was equivalent to more than 4000 individuals in each interval [38].

Two other studies from Germany reported higher prevalences in females of up to 18 years of age: boys (0.66%), girls (0.76%) [39]; boys (0.35%), girls (0.44%) [40]. The last one comprised 4499 children and adolescents. Another study from Spain found a prevalence of 0.27% for boys and 0.33% for girls in 217 patients under 18 years of age [41].

In four studies on childhood psoriasis, gender did not influence the mean age of onset [42,43,44,45]. The age of onset was earlier in boys only in 3 studies: girls 9.3 ± 2.3 years and boys 8.1 ± 2.1 years in 419 cases [46]; girls 13.8 years and boys 12.8 years in 59 pediatric patients [47]; girls 8.77 years and boys 7.00 years in 108 psoriatic patients under 18 years of age [48].

Immune factors involved in psoriasis (principally T-helper 17 and T-helper 1) are diminished in a high-estrogenic state such as pregnancy [49]. On the other hand, estrogen promotes keratinocyte proliferation and therefore induces epidermal hypertrophy. It is unknown which factors trigger its action from an anti-inflammatory function to a pro-inflammatory one [50]. Serum estrogen levels vary widely from 30 to 800 pg/mL during the menstrual cycle, while during pregnancy, they reach values around 20,000 pg/mL [50]. It has been suggested that high levels of estrogen would have an inhibitory effect on many components of the immune response while low levels could stimulate it [51]. It has also been observed that women with persistent irregular menstrual cycles would have a moderately higher risk of developing psoriasis [52]. As can be deduced, the fluctuations in estrogen levels are too large, and it has not been determined from what values they would have a protective effect on the development of psoriasis.

If the elevation of estrogen levels really has an immunomodulatory effect, inhibiting inflammatory processes or suppressing the autoimmune responses involved in the pathogenesis of psoriasis [1], one might wonder why psoriasis onset notably predominates in girls as described above, precisely at the stage (puberty) when this elevation begins to manifest itself. Could it be attributable to an indefinite fluctuation in estrogen levels around puberty or a varying estrogen receptor sensitivity or immaturity, perhaps acting on a predisposed ground? The latter hypothesis considers that in some studies, the frequency of familial affectation was higher in women with an early onset of psoriasis with respect to males [7,53,54]. It has been suggested that genetic factors have a stronger effect in early-onset psoriasis in young female patients with few other environmental influences for the disease [54]. If the psoriasis of early onset in girls was inherited, did it come from the father or the mother? Is it relevant?

In another aspect, could other hormones be involved or acting together? Progesterone levels alone apparently do not influence the improvement of psoriasis [55], while the elevation of cortisol associated with the elevation of estrogen does [52]. On the other hand, some increased adiposity is relatively common in boys and girls around puberty before they reach their growth spurt; just as there are also clear links between psoriasis and obesity, which may affect its severity or onset through the action of pro-inflammatory adipokines. It has been noted that obese children are at higher risk of developing psoriasis, so as a significant association between childhood psoriasis and being overweight or with abdominal obesity [56]. In this regard and just to mention it, an association between overweight and adolescence was observed in one study, with an increase in body mass index only in females with psoriasis [57].

Stress, another factor that frequently triggers or aggravates psoriasis in 26–88% of patients [58], would supposedly be the same for both sexes, even though puberty—a period of wide life transitions—occurs before in girls. If persistent, stress may lead to a decrease in cortisol levels, which may result in the upregulation of inflammatory cytokines [58] and also sleep disturbances. In addition, attention-deficit/hyperactivity disorder—a childhood-onset neurodevelopmental disorder characterized by inattention, hyperactivity, and impulsivity—has been strongly linked with psoriasis in females, despite being a male-predominant disorder with a ratio of 3:1 during childhood and adolescence [59]. Other environmental stressors, such as school, social or family requirements expected for a future young lady -added to hormonal fluctuations-, could interact in the development of psoriasis. However, these are difficult to quantify.

In summary, there are a lot of unresolved questions about the predominant onset of psoriasis in girls around puberty. Its possible relationship with estrogen levels during this period is not clear, being perhaps contradictory. Certainly, there must be some factor that determines the early onset of psoriasis in girls between the ages of 12 and 18 years and perhaps this interval could be better defined or specifically adjusted to puberty (approximately 10–15 years, possibly coinciding with menarche). Future studies on psoriasis during childhood/adolescence should ideally be more homogeneous or standardized, including basic detailed data for both sexes, such as age of onset (in short age intervals of much less than 10 years, at least during puberty) and a detailed history of familial affectation (psoriasis in siblings, paternal or maternal lineage, and their ages at onset). It would also be essential to evaluate the presence or absence of overweight (body mass index, waist circumference, or another metric) and routinely request basic tests to rule out a possible underlying metabolic syndrome. If estrogen determinations are performed, perhaps what role androgens and cortisol levels might play, if any, should be assessed.

## Figures and Tables

**Table 1:** Publications describing a higher frequency of psoriasis in girls or an early age of onset of psoriasis in females (y: years; **NR**: not reported; **NS**: not significant).

Ref.	Year	Country	Nº of Cases	Ages (y)	Frequency (%)		Average Age of Onset (y)		*p*
				Childhood/ Adolescence	Males	Females	Males	Females	
[3]	1906	USA	158	0–15	36.7	63.3			NR
[2]	1951	USA	147	0–19	41.5	58.5			NR
[4]	1966	USA	268	0–15	33.0	67.0			NR
[5]	1971	USA	777	0–19	36.0	64.0			NR
[6]	1975	Denmark	245	0–16	36.0	64.0	8.5	7.8	NR
[7]	1975	UK	419	<20	39.5	60.5			NR
[8]	1994	Kuwait	190	0–12	40.0	60.0			NR
[9]	2000	USA	223	<16	44.4	55.6			NR
[10]	2001	Australia	1262	0–15	47.0	53.0			NR
[11]	2006	Turkey	61	2–18			7.03 + 4.28	6.81 + 4.11	NR/NS
[12]	2007	China	277	0–15	46.9	53.1			NR
[13]	2010	China	137	0–14			11.4	8.9	NR
[14]	2011	Turkey	537	<18	39.5	60.5			0.36
[15]	2011	Asian	315	<16	41.9	58.1			NR
[16]	2013	Korea	1458	0–19	41.4	58.6			NR
[17]	2013	USA	181	5–17	40.3	59.7			NR
[18]	2017	Denmark	1925	<18	45.3	54.7			NR
[19]	2021	Thailand	177	0–10	44.1	55.9			NR
				**All Ages**					
[20]	1974	USA	5600	1–82			27.0	23.0	NR
[21]	1977	India	162	3–71			26.0	21.0	NR
[22]	1978	Sri Lanka	1286	0–88			27.0	16.5	<0.001
[23]	1985	USA	1511	0–40			22.0	16.0	NR
[24]	1986	India	782	NR			36.9 + 15.1	29.34 + 15.1	<0.05
[25]	1988	Spain	2523	0–89			31.68	26.59	<0.0001
[26]	1997	India	1220	0–80			30.9	27.6	NR
[27]	2002	Turkey	329	9–89			28 + 15	25 + 16	NR
[28]	2002	China	1043	0–79			27.69 + 12.32	23.26 + 12.56	<0.01
[29]	2002	Spain	1774	0 > 81			31.8 + 0.4	28.2 + 0.6	=0.001
[30]	2003	Japan	28,628	0–97			40.5	36.6	NR
[31]	2007	Italy	676	<30			20.6 + 6.6	19.1 + 8.7	=0.01
[32]	2010	Nederland	1926	18–90			26.8	24.3	<0.001
[33]	2011	Chile	153	9–79			29.2	24.8	NR/NS
[34]	2018	Malaysia	15,794	>18			37.09 + 15.5	32.59 + 16.6	NR
[35]	2020	Egypt	2534	1–94			32.7	28.1	0.001
[36]	2022	Malaysia	3932	1 > 70			42.0		<0.001
